# Patient-derived mouse models of cancer need to be orthotopic in order to evaluate targeted anti-metastatic therapy

**DOI:** 10.18632/oncotarget.12322

**Published:** 2016-09-28

**Authors:** Yukihiko Hiroshima, Ali Maawy, Yong Zhang, Nan Zhang, Takashi Murakami, Takashi Chishima, Kuniya Tanaka, Yasushi Ichikawa, Michael Bouvet, Itaru Endo, Robert M. Hoffman

**Affiliations:** ^1^ AntiCancer, Inc., San Diego, CA, USA; ^2^ Department of Surgery, University of California San Diego, San Diego, CA, USA; ^3^ Yokohama City University Graduate School of Medicine, Yokohama, Japan

**Keywords:** patient-derived orthotopic xenograft, PDOX, cervical cancer, nude mouse, primary tumor

## Abstract

Patient-derived xenograft (PDX) mouse models of cancer are emerging as an important component of personalized precision cancer therapy. However, most models currently offered to patients have their tumors subcutaneously-transplanted in immunodeficient mice, which rarely metastasize. In contrast, orthotopic-transplant patient-derived models, termed patient-derived orthotopic xenografts (PDOX), usually metastasize as in the patient. We demonstrate in the present report why orthotopic models are so important for the patient, since primary and metastatic tumors developed in an orthotopic model can have differential chemosensitivity, not detectable in standard subcutaneous tumor models. A subcutaneous nude mouse model of HER-2 expressing cervical carcinoma was not sensitive to entinostat (a benzamide histone deactylase inhibitor), which also did not inhibit primary tumor growth in a PDOX model of the same tumor. However, in the PDOX model, entinostat alone significantly reduced the metastatic tumor burden, compared to the control. Thus, only the PDOX model could be used to discover the anti-metastatic activity of entinostat for this patient. The results of the present report indicate the importance of using mouse models that can recapitulate metastatic cancer for precisely individualizing cancer therapy.

## INTRODUCTION

Patient-derived xenograft (PDX) mouse models of cancer are emerging as an important potential aid to personalized cancer therapy. However, most models offered are subcutaneously (s.c.) transplanted patient tumors in immunodeficient mice, which rarely metastasize [1]. Discrepancies have been described for decades between the invasive and metastatic behavior of tumors in the patient compared to their benign behavior in the s.c.-transplanted xenografts in immunocompetent mice [2]. More precise mouse models must be offered to the patient.

Wang and Sordat *et al*. [3] in 1982 were among the first to implant human tumors orthotopically (literally ‘correct place’) in nude mice, using cancer-cell suspensions, rather than “heterotopically” (literally “different place,” such as s.c.) [3]. Metastases as well as local tumor growth occurred in the orthotopic model, a very important advance. Our laboratory pioneered the patient-derived orthotopic xenograft (PDOX) nude mouse model with the technique of surgical orthotopic implantation (SOI) of intact colon cancer tissue [4, 5]. A greater extent of metastasis was observed in orthotopic models with implanted intact tumor tissue compared with orthotopically-implanted cell suspensions of the same tumor [6]. This perhaps is due to the intact histology and cancer-cell stroma interaction of the orthotopically-implanted tumor tissue.

PDOX models from patients with colon [4], pancreatic [7], breast [8], ovarian [9], lung [10], stomach cancer [11], and mesothelioma [12] were established in the early 1990s in our laboratory, resulting in primary and metastatic tumor growth very similar to that of the patient [11]. Recently, PDOX models of sarcoma have been developed [13–15] as well as additional models of pancreatic cancer PDOX [16–19] and colon cancer PDOX [5, 20].

We have also recently described the development of a PDOX model of HER2-positive cervical cancer. Metastasis in nude mice included peritoneal dissemination, liver metastasis, lung metastasis, as well as lymph node metastasis, reflecting the metastatic pattern in the donor patient. Primary tumors and metastases in the nude mice had histological structures similar to those in the original tumor and were stained by a HER2-specific antibody in the same pattern as was the patient's original cancer [21].

In the present report, we describe that the benzamide histone deacetylase inhibitor, entinostat, was not active in a subcutaneous PDX nude mouse model of the HER-2 expressing cervical carcinoma described above, nor did entinostat prevent primary tumor growth in the PDOX model of the same tumor. In contrast, in the PDOX model, entinostat significantly reduced the metastatic tumor burden compared to the control. Thus, only the PDOX model could be used to discover the anti-metastatic activity of entinostat for this patient. The results of the present report indicate the importance of using patient-derived mouse models that can recapitulate metastatic cancer for precise personalized therapy.

## RESULTS AND DISCUSSION

**Figure 1:**
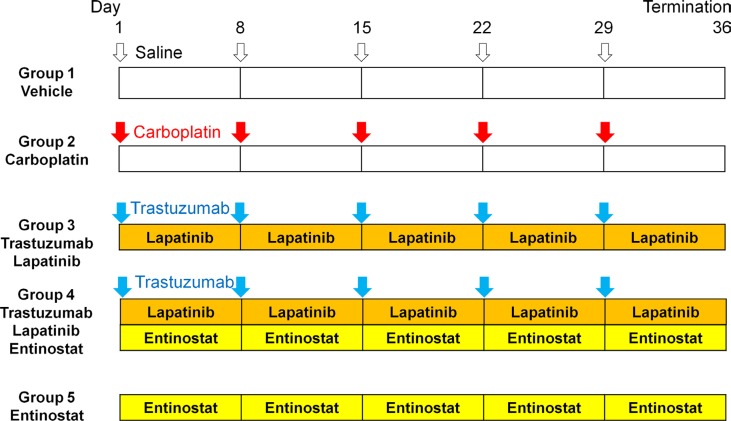
Schema of experiments The patient HER-2-expressing squamous carcinoma of the cervix was established subcutaneously in nude mice. Small fragments of subcutaneously-growing tumor were harvested, minced and orthotopically implanted in the cervix of 25 nude mice. Six weeks after implantation, the mice in each model were randomized and treated in the following groups of *n* = 5: (1) saline (control, ip, weekly, 5 weeks); (2) carboplatin (30 mg/kg, ip, weekly, 5 weeks); (3) trastuzumab (20 mg/kg, ip, weekly, 5 weeks) + lapatinib (100 mg/kg, orally, daily, 5 weeks); (4) trastuzumab (20 mg/kg, ip, weekly, 5 weeks) + lapatinib (100 mg/kg, orally, daily, 5 weeks) + entinostat (5 mg/kg, orally, daily, 5 weeks); and (5) entinostat (5 mg/kg, orally, daily, 5 weeks).

Treatment protocols are outlined in Figure 1.

### Entinostat did not arrest tumor growth in the subcutaneous model of patient cervical cancer

**Figure 2:**
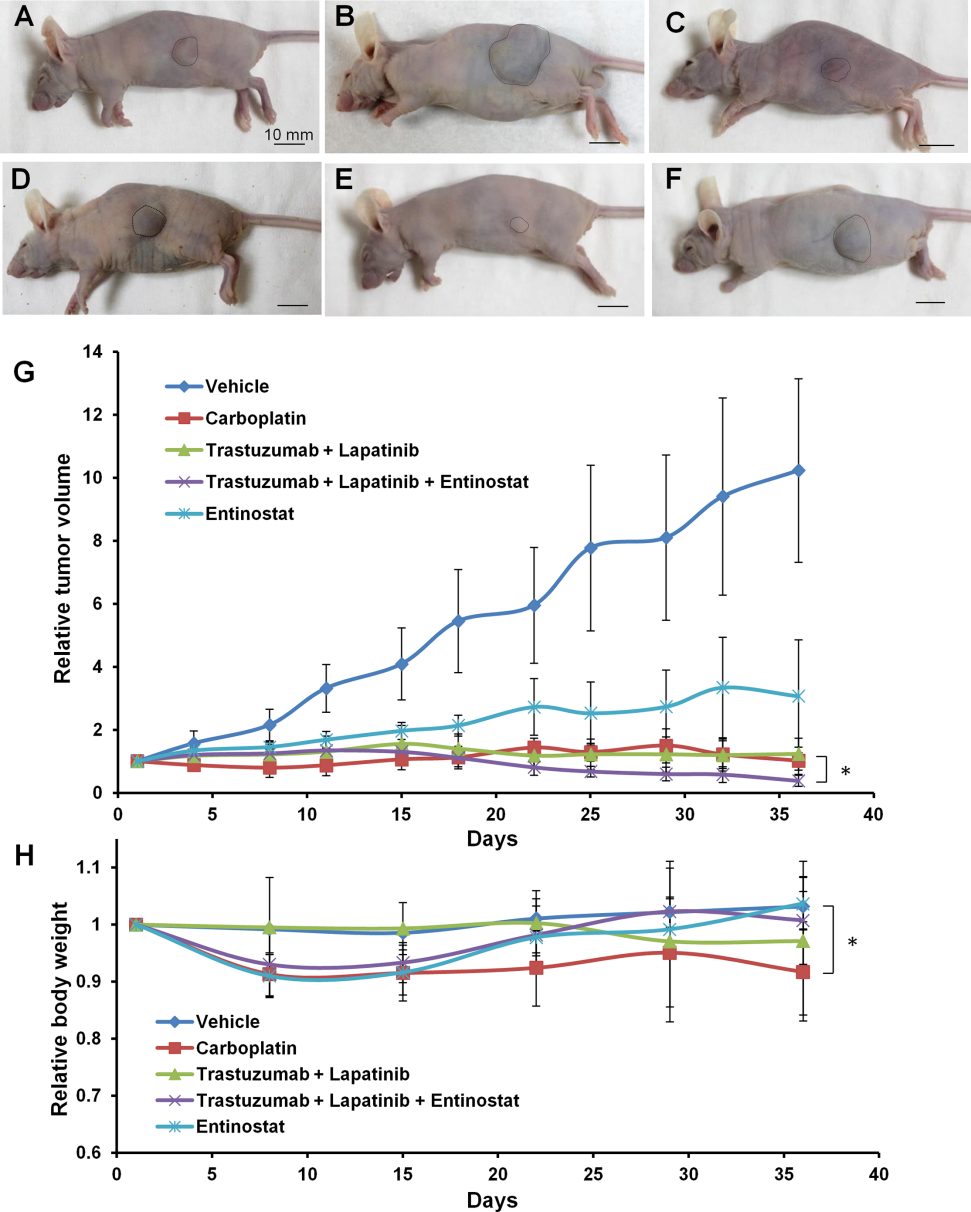
Drug-efficacy testing in the subcutaneous tumor model Representative images of nude mice with subcutaneous tumor before treatment (**A**), or treated with saline (**B**), carboplatin (**C**), trastuzumab + lapatinib (**D**), trastuzumab + lapatinib + entinostat (**E**) or entinostat (**F**). The areas surrounded by black broken lines indicate the subcutaneous tumors. Scale bar: 10 mm. (**G**) Growth curves of the subcutaneous PDXs treated with various drugs as described above. (*n* = 5 for each treatment arm). (**H**) Body weight curves of the mice with the subcutaneous PDXs treated with various drugs.

Entinostat monotherapy was least effective compared to carboplatin, trastuzumab and lapatinib in the subcutaneous nude mouse model of patient cervical cancer (Figure 2).

### Entinostat monotherapy was not active on the primary tumor of the PDOX model of cervical cancer

**Figure 3:**
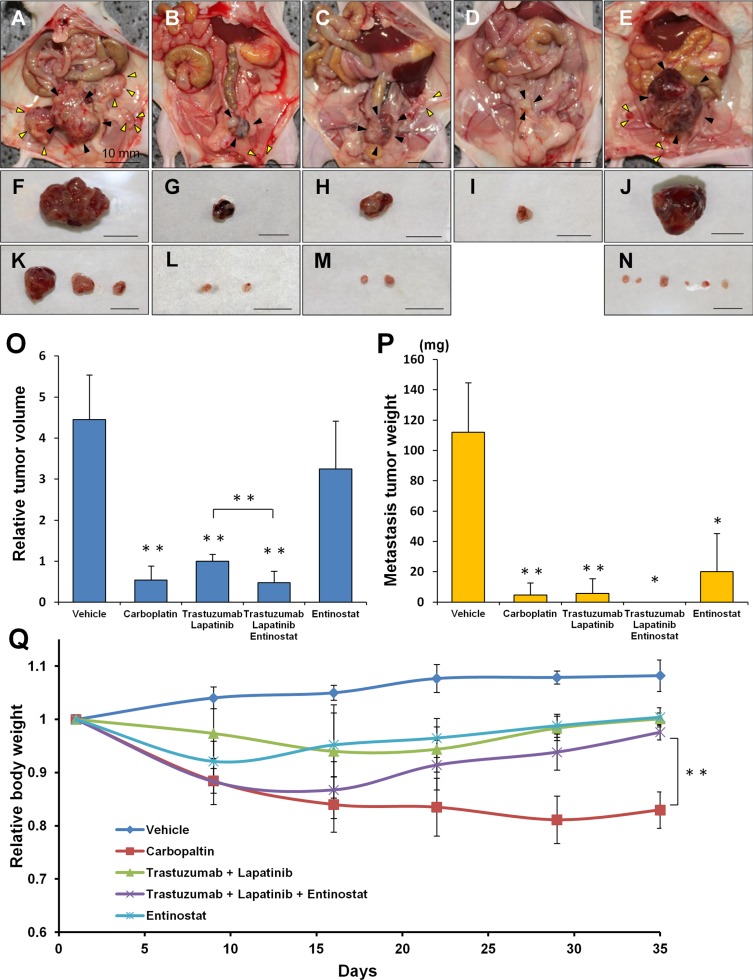
Drug-efficacy testing in the PDOX model Representative images of laparotomy of the nude mice with the PDOX treated with saline (**A**), carboplatin (**B**), trastuzumab + lapatinib (**C**), trastuzumab + lapatinib + entinostat (**D**), or entinostat (**E**). Black arrow heads or yellow arrow heads indicate primary tumors or peritoneally-disseminated tumors, respectively. (**F**–**J**) Excised specimens of primary tumors corresponding to Figures A–E, respectively. (**K** –**N**) Excised specimens of peritoneally-disseminated tumors corresponding to Figures A–E, respectively. Scale bar: 10 mm. (**O**) Bar graphs of the primary tumor weight in each group. (**P**) Bar graphs of the metastatic tumor weight in each group. (*n* = 5 for each treatment arm). (**Q**) Body weight curves of the mice with the PDOXs treated with various drugs. *p < 0.05, **p < 0.01.

All regimens tested except entinostat had significant efficacy on the primary tumors compared to the vehicle control group: carboplatin, *P* = 0.004; trastuzumab/lapatinib, *P* = 0.03; trastuzumab/lapatinib/entinostat, *P* < 0.001; respectively. Entinostat monotherapy compared to vehicle control, *P* = 0.262 (Figure 3).

### Entinostat monotherapy was active against metastasis in the PDOX model of cervical cancer

However, entinostat had efficacy against metastasis in the PDOX model of cervical carcinoma. All other regimens tested also had significant efficacy on metastasis compared to the vehicle group: carboplatin, *P* = 0.005; trastuzumab/lapatinib, *P* = 0.006; trastuzumab/lapatinib/entinostat, *P* = 0.027; entinostat, *P* = 0.018; respectively (Figure 3). No metastasis was detected in the trastuzumab/lapatinib/entinostat group (Figure 3). All regimens caused body-weight loss, with carboplatin the most toxic (Figure 3).

### Histology

**Figure 4:**
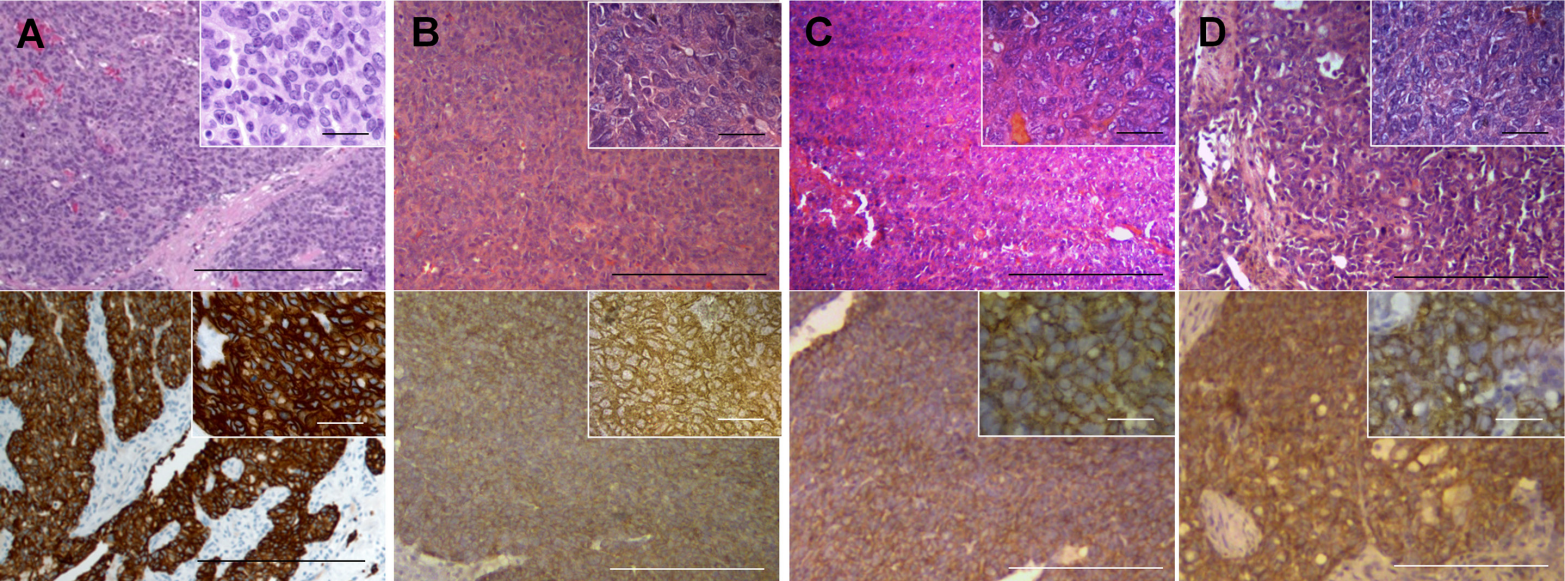
Histology of the patient and mouse-grown tumors and metastasis H&E-stained and immunostained sections of mouse-grown tumors. Upper panels are H&E-stained sections and lower panels are immunostained sections using an anti-HER-2 antibody. Right upper insets are high-magnification images (scale bars: 25 μm). All mouse-grown tumors, including the subcutaneous tumors (**A**), primary orthotopic tumor (**B**), peritoneal-disseminated metastasis (**C**) and liver metastasis (**D**) had histological structures similar to the original patient tumor and were stained by an anti-HER-2 antibody. Scale bars: 200 μm (A–D, lower magnification) [21].

We previously examined the histology of the PDOX model using H&E staining and immunostaining with anti-HER2 antibody [21]. Sheet-like growth without gland formation fibroblastic cells, which penetrated into the nests of carcinoma, were found in the H&E stained sections of the original tumor. A range of oval- to spindle-shaped cancer cells with high nuclear/cytoplasmic ratios were observed in high-magnification images. Immunohistochemistry with an anti-HER-2 antibody showed that the membrane and the cytoplasm of cancer cells was strongly stained but no staining was found in the stromal tissue. The mouse-grown primary and metastatic tumors had histological structures similar to the original tumor and were stained by anti-HER-2 antibody (Figure 4B–4D) [21].

## MATERIALS AND METHODS

### Animals

Female athymic *nu/nu* nude mice (AntiCancer Inc., San Diego, CA), 4–6 weeks old, were used in this study. Mice were kept in a barrier facility under HEPA filtration. Mice were fed with autoclaved laboratory rodent diet. All mouse surgical procedures and imaging were performed with the animals anesthetized by intramuscular injection of a 0.02 ml solution of 50% ketamine, 38% xylazine, and 12% acepromazine maleate. All animal studies were conducted with an AntiCancer Institutional Animal Care and Use Committee (IACUC)-protocol specifically approved for this study and in accordance with the principals and procedures outlined in the National Institute of Health Guide for the Care and Use of Animals under Assurance Number A3873-1.

### Patient consent and IRB approval

The patient with HER-2-expressing cervical cancer provided informed consent and the study was conducted under the approval of the Institutional Review Board (IRB) of the UC San Diego Medical Center.

### Establishment of patient-derived cervical cancer

Tumor tissues were obtained from the HER-2-positive cervical cancer patient at surgery, divided into 3-mm^3^ fragments, and transplanted subcutaneously in nude mice [21].

### Orthotopic tumor implantation

After the subcutaneous tumors grew in the nude mice, they were harvested and divided into small fragments for orthotopic transplantation which was performed as follows: a small 6− to 10-mm midline incision was made on the lower abdomen of the nude mouse through the skin and peritoneum. The uterus was exposed through this incision, and a single 3-mm^3^ tumor fragment was sutured to the cervix of the uterus using 8–0 nylon surgical sutures (Ethilon; Ethicon Inc., NJ, USA). On completion of tumor implantation, the uterus was returned to the abdomen, and the incision was closed in one layer using 6–0 nylon surgical sutures (Ethilon) [21].

### Treatment

Six weeks after implantation, the mice in the PDX and PDOX models were randomized and treated in the following groups of *n* = 5: (1) saline control, (ip, weekly, 5 weeks); (2) carboplatin (Selleck Chemicals, Houston, TX, USA, 30 mg/kg, ip, weekly, 5 weeks); (3) trastuzumab (Genentech, Inc., South San Francisco, CA, USA, 20 mg/kg, ip, weekly, 5 weeks) + lapatinib (Selleck Chemicals, 100 mg/kg, orally, daily, 5 weeks); (4) trastuzumab (20 mg/kg, ip, weekly, 5 weeks) + lapatinib (100 mg/kg, orally, daily, 5 weeks) + entinostat (Selleck Chemicals, 5 mg/kg, orally, daily, 5 weeks); and (5) entinostat (5 mg/kg, orally, daily, 5 weeks) (Figure 1). For the subcutaneous model, tumor size was evaluated every 3 or 4 days by caliper measurements and the approximate tumor volume was calculated using the formula 4/3π.(d/2)^2^. D/2, where d is the minor tumor axis and D is the major tumor axis.

For the orthotopic model, the mice underwent laparotomy 1 week before treatment to confirm the presence of the primary tumor, and its size was evaluated as described above.

Relative tumor volume and body weight were calculated by comparison to tumor size before treatment. Animals underwent laparotomy after treatment, and the tumors were photographed with a Canon EOS 60D digital camera with an EF–S18–55 IS lens (Canon, Tokyo, Japan) and weighed and harvested for analysis. Body weight of the mice was measured in a balance once a week.

### Tissue histology

Tumor samples were removed with surrounding normal tissues at the time of resection. Fresh tissue samples were fixed in 10% formalin and embedded in paraffin before sectioning and staining. Tissue sections (3 μm) were deparaffinized in xylene and rehydrated in an ethanol series. Hematoxylin and eosin (H&E) staining was performed according to standard protocols. For immunohistochemistry, the sections were then treated for 30 min with 0.3% hydrogen peroxide to block endogenous peroxidase activity. The sections were subsequently washed with PBS and incubated in citrate antigen unmasking solution (Mitsubishi Kagaku Iatron, Inc., Tokyo, Japan) in a water bath for 40 min at 98°C. After incubation with 10% normal goat serum, the sections were incubated with anti-HER-2/ErbB-2 antibody (1:100, Cell Signaling Technology, Danvers, MA, USA) at 4°C overnight. The binding of primary antibodies was detected using anti-mouse secondary antibodies and avidin/biotin/horseradish peroxidase complex (DAKO Cytomation, Kyoto, Japan) for 30 min at room temperature. The labeled antigens were visualized with the DAB kit (DAKO Cytomation). Finally, the sections were counterstained with hematoxylin and examined using an Olympus BH-2 microscope equipped with an INFINITY1 2.0 megapixel CMOS digital camera (Lumenera Corporation, Ottawa, Canada). All images were acquired using INFINITY ANALYZE software (Lumenera Corporation) without post-acquisition processing (Figure 4).

### Statistical analysis

PASW Statistics 18.0 (SPSS, Inc.) was used for all statistical analyses. The Student's *t*-test was used to compare continuous variables between two groups. Analysis of variance models were used to compare multiple groups. A *P*-value of 0.05 was considered statistically significant for all comparisons.

## CONCLUSIONS

The PDOX model recapitulated the metastatic potential of the original cervical-cancer patient tumor (Figure 3) [21]. Most importantly, entinostat monotherapy significantly inhibited the metastasis of HER-2-positive cervical cancer even though there was no efficacy of this agent on the primary tumor or on the subcutaneous model. The efficacy of entinostat would not have been detected in a subcutaneous PDX model of this tumor.

Patient-derived mouse models of cancer are an emerging field for individualized precise treatment of cancer. Although such models were first described in 1969 [2], patient-derived models are now recognized for their potential in individualized, precision cancer treatment. The present report indicates the importance of orthotopic mouse models for their potential to most precisely represent the cancer patient for individualized therapy, most importantly to evaluate targeted anti-metastatic therapy. Future studies will measure tumor growth as a 3-dimensional geometric mean to better determine logarithmic growth.

Previously-developed concepts and strategies of highly-selective tumor targeting can take advantage of molecular targeting of tumors, including tissue-selective therapy which focuses on unique differences between normal and tumor tissues [22–27].
